# Malignant hyperthermia: a review

**DOI:** 10.1186/s13023-015-0310-1

**Published:** 2015-08-04

**Authors:** Henry Rosenberg, Neil Pollock, Anja Schiemann, Terasa Bulger, Kathryn Stowell

**Affiliations:** Department of Medical Education and Clinical Research, Saint Barnabas Medical Center, Livingston, NJ 07039 USA; Department of Anesthesia and Intensive Care, Palmerston North Hospital, Palmerston North, New Zealand; Institute of Fundamental Sciences, Massey University, Palmerston North, New Zealand

**Keywords:** Malignant Hyperthermia, Anesthesia, Ryanodine receptor

## Abstract

Malignant hyperthermia (MH) is a pharmacogenetic disorder of skeletal muscle that presents as a hypermetabolic response to potent volatile anesthetic gases such as halothane, sevoflurane, desflurane, isoflurane and the depolarizing muscle relaxant succinylcholine, and rarely, in humans, to stressors such as vigorous exercise and heat. The incidence of MH reactions ranges from 1:10,000 to 1: 250,000 anesthetics. However, the prevalence of the genetic abnormalities may be as great as one in 400 individuals. MH affects humans, certain pig breeds, dogs and horses. The classic signs of MH include hyperthermia, tachycardia, tachypnea, increased carbon dioxide production, increased oxygen consumption, acidosis, hyperkalaemia, muscle rigidity, and rhabdomyolysis, all related to a hypermetabolic response. The syndrome is likely to be fatal if untreated. An increase in end-tidal carbon dioxide despite increased minute ventilation provides an early diagnostic clue. In humans the syndrome is inherited in an autosomal dominant pattern, while in pigs it is autosomal recessive. Uncontrolled rise of myoplasmic calcium, which activates biochemical processes related to muscle activation leads to the pathophysiologic changes. In most cases, the syndrome is caused by a defect in the ryanodine receptor. Over 400 variants have been identified in the *RYR1* gene located on chromosome 19q13.1, and at least 34 are causal for MH. Less than 1 % of variants have been found in *CACNA1S* but not all of these are causal. Diagnostic testing involves the *in vitro* contracture response of biopsied muscle to halothane, caffeine, and in some centres ryanodine and 4-chloro-m-cresol. Elucidation of the genetic changes has led to the introduction of DNA testing for susceptibility to MH. Dantrolene sodium is a specific antagonist and should be available wherever general anesthesia is administered. Increased understanding of the clinical manifestation and pathophysiology of the syndrome, has lead to the mortality decreasing from 80 % thirty years ago to <5 % in 2006.

## Introduction

This review summarizes current diagnostic, management and treatment practices for the rare genetic disorder malignant hyperthermia in the context of the current understanding of the structure and function of the skeletal muscle calcium channel. This review is intended for a general audience with an interest in malignant hyperthermia from a clinical or biomedical perspective. The most common form of malignant hyperthermia can be triggered by volatile anesthetic agents and can be fatal if not treated promptly. Other relevant disorders and complications are also discussed. Of particular note are the recent advances in DNA based diagnosis with the advent of accessible genome sequence analysis. Problems associated with the widespread use of DNA-based diagnosis are highlighted. Finally, a section on unresolved issues highlights the complexity of malignant hyperthermia, the underlying genetics and the potential crosstalk with related disorders of calcium handling in skeletal muscle.

## Review

Disease name and synonyms

Malignant hyperthermia

Malignant hyperpyrexia

Hyperthermia of anesthesia

ORPHA423

### Definition

Malignant hyperthermia (MH) is a pharmacogenetic disorder that manifests as a hypermetabolic response to potent inhalation agents (such as halothane, isoflurane, sevoflurane, desflurane), the depolarizing muscle relaxant succinylcholine, and rarely, in humans, to stressors such as vigorous exercise and heat. The two genes that have been definitively associated with MH causative mutations are *RYR1* and *CACNA1S,* which will be discussed later.

As almost all patients who are MH susceptible have no phenotypic changes without anesthesia, it is impossible to diagnose susceptibility without either exposure to the “trigger” anesthetics or by specific diagnostic testing. The key clinical features include an unexplained elevation of expired carbon dioxide, despite increased minute ventilation, muscle rigidity and rhabdomyolysis, hyperthermia, tachcardia, acidosis and hyperkalemia. The majority of patients with Central Core Disease (CCD), an inherited myopathy characterized by muscle weakness, are susceptible to MH. Multi-minicore Disease (MmD), central nuclear myopathy and King-Denborough syndrome also predispose to episodes of MH.

### Epidemiology

The incidence of MH episodes during anesthesia is between 1:10,000 and 1:250,000 anesthetics [1, 2]. Even though an MH crisis may develop at first exposure to anesthesia with those agents known to trigger an MH episode, on average, patients require three anesthetics before triggering. Reactions develop more frequently in males than females (2:1) [3, 4]. All ethnic groups are affected, in all parts of the world. The highest incidence is in young people, with a mean age of all patients experiencing reactions of 18.3 years. It has been found that children under 15 years age comprised 52.1 % of all reactions [5]. Although described in the newborn, the earliest reaction confirmed by testing is six months of age [6]. The oldest is 78 years.

The estimated prevalence of genetic abnormalities associated with MH susceptibility may be as great as one in 3000 individuals (range 1:3000 to 1:8500), with a more recent estimate being 1 in 400 [7].

Mauritz et al. [8] found an incidence of 1:37,500 in patients who had been diagnostically tested, which was similar to the incidence estimated by Robinson et al. (1:30,000) [9] although wide variability has been reported. A recent report suggested that the MH susceptible (MHS) trait may be present in 1:2000–3000 of the French population [10]. A similar incidence was reported for the Japanese population [11]. Bachand and colleagues traced the pedigrees of MH patients in Quebec, Canada to the original immigrants from France and found an incidence of MH susceptibility of 0.2 % in this province. However, that represented only five extended families. Similarly 1/200 patients presenting for anesthesia in the Manawatu region of New Zealand are either susceptible or related to MHS individuals (unpublished data – N Pollock, T Bulger).

A study of 12 million hospital discharges in the state of New York demonstrated the prevalence of MH to be one in 100,000 surgical procedures although the type of anesthetic was not indicated. This likely represents an underestimate of MH in association with general anesthesia [4].

MH crises develop not only in humans but also in other species, particularly pigs, which have been a valuable source for research. Reactions have also been described in horses, dogs and other animals [12].

### Clinical description

MH may occur at any time during anesthesia as well as in the early postoperative period, but not after an hour of discontinuation of volatile agents [13]. The earliest signs are tachycardia, rise in end-expired carbon dioxide concentration despite increased minute ventilation, accompanied by muscle rigidity, especially following succinylcholine administration. Body temperature elevation can be a dramatic sign of MH. Larach et al. found that increased temperature was the first to third earliest sign in 63.5 % of MH reactions [14]. This confirms Sessler’s comment that core temperature should be monitored in most patients undergoing general anesthesia for periods lasting more than 30 min and in all patients with anesthesia lasting 60 mins [15].

Although end-tidal carbon dioxide (ETCO_2_) is a sensitive early sign of MH [16], in recent years, with a decline in the use of succinylcholine, rather than an abrupt rise in CO_2_, a more gradual rise is often noted. Indeed, by increasing minute ventilation it is possible to mask this rise [17].

Hyperthermia can be marked, with an increase in core temperature at a rate of 1–2 °C every five minutes. Severe hyperthermia (core temperature greater than 44 °C) may occur, and lead to a marked increase in oxygen consumption, CO_2_ production, widespread vital organ dysfunction, and disseminated intravascular coagulation (DIC) [18].

Uncontrolled hypermetabolism leads to respiratory and in most cases metabolic acidosis due to rapid consumption of energy stores and ATP. If untreated, continuing myocyte death and rhabdomyolysis result in life-threatening hyperkalemia; myoglobinuria may lead to acute renal failure. Additional life-threatening complications include DIC, congestive heart failure, bowel ischemia, and compartment syndrome of the limbs secondary to profound muscle swelling. Indeed, when body temperature exceeds approximately 41 °C, DIC is the usual cause of death.

### Rhabdomyolysis

Rhabdomyolysis refers to the breakdown of skeletal muscle, which is associated with excretion of myoglobin in the urine. Classically, MH presents with hypercarbia, tachycardia, cardiac arrhythmias, pyrexia, rigidity and metabolic acidosis, and rhabdomyolysis as a late sign. Several reports of isolated rhabdomyolysis apparent immediately following anesthesia or developing up to 24 h post anesthesia have been reported [19, 20]. Increased creatine kinase (CK) measurement and a positive *in vitro* contracture test (IVCT, covered in a subsequent section) have been obtained in these patients, indicating MH susceptibility. MH-like muscle responses however, can represent false positive diagnoses and an underlying myopathic process may produce a positive IVCT [21] so there must remain some doubt on the validity of this feature i.e., rhabdomyolysis as an expression of MH. Burns et al. stated however that MH should be considered in all patients presenting with rhabdomyolysis where the degree of muscle necrosis exceeds that expected for the severity of the accompanying disorder [22]. The most prudent diagnostic course, therefore, is contracture testing for MH susceptibility.

### Complications

A recent report from the North American Malignant Hyperthermia Registry (NAMHR) of the Malignant Hyperthermia Association of the United States (MHAUS) demonstrated that early recognition of the signs of MH and routine use of core temperature monitoring are essential in minimizing morbidity and mortality from MH. Larach and colleagues showed that in analyzing deaths from MH, in 8 of 84 patients the risk of dying from MH was about 14 times greater in those patients where core temperature monitoring was not used and 9.7 times greater where only skin temperature monitoring was used. The data also showed that the likelihood of any complication increased 2.9 times per 2^o^ C in maximum temperature and 1.6 times per 30 min delay in dantrolene use. Furthermore, the time interval between anesthetic induction to maximum ETCO_2_ was longer in cases with cardiac arrest/death compared with the others (216 *versus* 87 min) [23]. Other signs include acidosis, tachypnea and hyperkalemia. The progression of the syndrome may be rapid and dramatic, particularly if precipitated by succinylcholine, or more slowly and not become manifest until after several hours after induction of anesthesia.

### Pharmacological triggers

Numerous factors could be involved in triggering MH – age, type of anesthetic, environmental temperature, mitigating drugs administered simultaneously, genetic makeup and degree and type of stress [2].

All inhalation anesthetics except nitrous oxide are triggers for MH. The muscle relaxant succinylcholine is also a trigger for MH. No other anesthetic drugs appear to be triggers, including propofol and ketamine. Neither are catecholamines, nondepolarizing muscle relaxants, catechol congeners, digitalis or similar agents [24].

Another potential risk factor is the use of inhalational sedation devices postoperatively in the intensive care unit (ICU) for a range of different conditions [25–28]. Patients susceptible to MH also resident in the ICU may be at risk from such exposure, although administration of sevoflurane via the AnaConDa® device was found to be safe for healthcare workers with the caveat that a gas extraction system should be used in conjunction with such devices to reduce occupational exposure [29]. A case of MH triggered by sevoflurane administration via an AnaConDa® was reported in a patient admitted to ICU for lumbalgia. MH susceptibility was confirmed at a later date, highlighting the significance of MH differential diagnosis in intensive care patients admitted for other conditions, if these types of sedation devices are used [30].

### Disorders associated with malignant hyperthermia

Succinylcholine induced masseter muscle rigidity (MMR) occurs in 1 in 100 children with anesthesia induced by halothane and given succinylcholine [31]. The incidence is probably the same following induction with sevoflurane, but much less following induction with thiopental [32]. The clinical incidence of MH as defined by arterial blood gas changes is about 15 % after MMR. However, muscle biopsy reveals that 50 % of patients experiencing MMR are MH susceptible [33]. Patients with generalized rigidity along with MMR are at much greater risk for MH. Kaplan (personal communication,) has hypothesized that children with “jaws of steel” as opposed to mild rigidity after administration are at greater risk for MH. He has hypothesized that the children with the more dramatic masseter rigidity are more often referred for biopsy and hence the high incidence of positive biopsies.

Central Core Disease (CCD) is a rare non-progressive myopathy with mainly autosomal dominant inheritance, presenting in infancy and characterized by hypotonia and proximal muscle weakness. A few families demonstrate autosomal recessive inheritance. Histological examination of affected muscles shows a predominance of type I fibres containing clearly defined areas (cores) lacking oxidative enzyme activity [34–36].

CCD patients are often susceptible to MH as confirmed by accepted muscle biopsy caffeine-halothane contracture testing (either IVCT or the CHCT-caffeine halothane contracture test – see laboratory diagnostic methods section), but MH and CCD phenotypes do not always co-segregate within families. Patients with MH may present with cores despite being clinically asymptomatic and with some *RYR1* variants (specifically some of those in the C-terminal transmembrane domain of the protein) specific to CCD. DNA Sequencing showed that *RYR1* variants occurred in over 93 % (25 out of 27) of Japanese patients with CCD [37]. While this is of importance, it may not reflect the incidence of *RYR1* mutations in other populations. Another study indicated that the distribution and frequency of *RYR1* variants differed markedly in the Japanese MH susceptible population as compared to the North American and European MH susceptible population [11]. Although *RYR1* variants are the most common identified cause of CCD, it does show genetic heterogeneity, with several rare susceptibility loci known (the *ACTA1* gene, in association with nemaline myopathy, and the *MYH7* gene, in association with hypertrophic cardiomyopathy), with further loci yet to be identified [38].

Other myopathies that have been suggested to be associated with MH susceptibility include MmD and centronuclear myopathy. MmD is an early onset congenital myopathy that may affect bulbar, respiratory and extraocular muscles and has autosomal recessive inheritance [39]. Recessive variants in *RYR1* have been associated with MmD, some of which result in altered Ca^2+^ release from intracellular stores and others that do not [40]. Taken together, these observations suggest that there may be a subset of *RYR1* variants that result in both MH and MmD and a subset that are associated only with MmD, similar to the situation with MH and CCD. Consequently, it will be important to distinguish between *RYR1* variants that result in MmD, and those that do not.

King (or King-Denborough) syndrome [41] is a rare myopathy characterized by dysmorphic facies, ptosis, down-slanting palpebral fissures, hypertelorism, epicanthic folds, low-set ears, malar hypoplasia, micrognathia, high-arched palate, clinodactyly, palmar simian line, pectus excavatum, winging of the scapulae, lumbar lordosis and mild thoracic scoliosis. The patients with King-Denborough syndrome also present congenital hypotonia, slightly delayed motor development, diffuse joint hyperextensibility and mild proximal weakness. Such patients are MH susceptible. Gillies et al. identified a causative mutation in one family affected with King-Denborough syndrome [42]. Dowling however, did not find a causative mutation to be a consistent feature in this syndrome [43].

### Etiology

MH is considered to be a pharmacogenetic disorder which results in a hypermetabolic state [44]. Experimental evidence clearly indicates that the signs and symptoms of MH are related to an uncontrolled release of intracellular Ca^2+^ from skeletal muscle sarcoplasmic reticulum (SR) [45]. In MH susceptible swine and in “knock-in” mice, a variety of environmental conditions can trigger accelerated Ca^2+^ release from the SR such as environmental heat, exercise and stress. In humans, however, clinical MH results most often from exposure to potent inhalation anesthetics +/− succinylcholine. The enhanced intracellular Ca^2+^ results in abnormal skeletal muscle metabolism manifesting as activation of muscle contraction, increased oxygen consumption and CO_2_ production, ATP hydrolysis and heat production. The normal sequestration of released Ca^2+^ by the SR/ER Ca^2+^ -ATPase (SERCA) is inadequate and energy is expended in a futile manner, in an attempt to lower intracellular Ca^2+^. Presumably, the declining levels of ATP lead to failure of membrane integrity and release of potassium and CK, although the exact steps in the process have not been definitively demonstrated.

A defective or disordered Ca^2+^ channel located in the SR membrane underlies MH susceptibility. This channel is termed the ryanodine receptor (RyR1). As many as 70 % of families susceptible to MH harbor one of 34 causal mutations for MH, with many other variants yet to be characterized [46]. The channel is closely associated with many other proteins, such as the dihydropyridine receptor (DHPR) Ca^2+^ channel, situated in the T-tubule region of the sarcolemma that mediates transfer of voltage change to the RyR1 receptor. Other proteins with potential or known roles in RyR1 function include integral SR membrane proteins (eg. SRP-27 [47], junctate [48], the transient receptor potential cation channel (TRPC) family [48–50] and triadin [51]), plasma membrane-associated proteins (eg. CIC-1 chloride channels [52] and Na^+^/Ca^2+^ exchangers [53]), as well as proteins that appear to have a role in stabilizing the junction between the plasma membrane and sarcoplasmic reticulum (eg. junctophilin and caveolin-3) by interacting with both DHPR and RyR1 [54]. Proteins that modulate the function of RyR1 include the FK508 binding protein FKBP12 [55], the Ca^2+^ binding protein calmodulin [56], the histidine-rich Ca^2+^ protein, HRC [57] and the luminal Ca^2+^ buffer calsequestrin. HRC is also a luminal protein known to interact with both triadin and SERCA and has been suggested to have a role in mediating cross talk between SR Ca^2+^ uptake and release [57].

At least six genetic loci, other than *RYR1* have been implicated in MH, although only one other gene, *CACNA1S*, encoding the main subunit of the DHPR, has been shown to be altered by an MH-linked variant [58–60]. Calsequestrin has been suggested as another candidate for MH from studies using a *CASQ1* knock-out mouse [61–63]. These mice exhibited susceptibility to heat- and anesthetic-induced mortality, analogous to MH. While some *CASQ1* variants have been identified in humans [64], there is thus far no definitive evidence that variants in this gene can cause MH [65]. Recently, a variant in the *STAC3* gene has been linked to MH susceptibility in a native American tribe in the USA [66]. Ablation of *stac3* in Zebrafish results in a severe locomotor defect and a decrease in excitation-contraction coupling [67]. *STAC3* knock-out mice exhibit paralysis and perinatal lethality as well as a range of musculoskeletal defects [68]. In support of a role in excitation-contraction coupling, the STAC3 protein was shown to traffic together with the DHPR and has been suggested to be an essential chaperone of DHPR in skeletal muscle [69].

JP-45, encoded by *JSPR1*, is another integral SR protein that has been shown to colocalize with the RyR1 and also interacts with the DHPR and calsequestrin. Overexpression of JP-45 in a mouse myotube cell line has been shown to decrease charge movement through the DHPR. Depletion of JP-45 in the same system decreased both the content of DHPR and charge movement through this channel [70]. Two *JSPR1* variants have been recently identified in patients with and without MH. Expression of either one of these JP-45 variants in mouse muscle fibres exhibited a decrease in the sensitivity of DHPR to activation. These results suggest that the overall phenotype of an individual with both a *JSPR1* mutation and a causative *RYR1* mutation would be less severe than if the *RYR1* mutation was expressed alone [71]. These observations highlight the possibility of polymorphic variants modulating *RYR1* function and may help to explain the variable phenotype observed for MH susceptibility [9, 72].

Genotype-phenotype correlations are weak for both the clinical expression of MH and the response of isolated muscle to caffeine or halothane. It therefore seems clear that a variety of modulators influence the manifestations of the syndrome. Fatty acids represent one set of modulators that has been studied in this respect [73, 74]. Certain unsaturated fatty acids have been demonstrated to increase the sensitivity of halothane-induced Ca^2+^ release *in vitro*. Such an increase in fatty acids may result from breakdown of triglycerides as a result of enzymatic abnormalities. More recently, a decrease in S-palmitoylation at cysteine residues in the N-terminal region of RyR1 has been shown to decrease stimulus-coupled Ca^2+^ release via RyR1 [75]. Ryanodine receptor function can also be altered by other post-translational modifications. Phosphorylation, glutathionylation, oxidation and nitrosylation of *RyR1* have each been shown to modulate Ca^2+^ release from the SR, but the causes and functional consequences of these modifications are not well defined [76–80]. Eight of the eighteen cysteine residues subject to S-palmitoylation are also targets for N-nitrosylation or S-oxidation, suggesting that post-translational cross-talk may have a role in regulating RyR1 [75]. SERCA and the DHPR are also subject to S-palmitoylation suggesting that fatty acids may have more extensive roles in excitation-contraction coupling and hence MH.

In addition, cultured muscle cells from MH susceptible patients show a shift of subtypes of sodium channels leading to a longer membrane depolarization and an increased Ca^2+^ release from the terminal cisternae [81, 82]. Changes in sodium channel function, either through sodium channel mutations or through effects of fatty acids may influence the phenotypic expression of MH, especially muscle rigidity.

Ca^2+^ depletion of the SR via skeletal muscle RyR1 activity has also been shown to induce Ca^2+^ influx across the plasma membrane. Both store-operated Ca^2+^ entry (SOCE) and excitation-coupled Ca^2+^ entry (ECCE) are involved [83–85]. While the exact mechanisms that control these phenomena are unclear, membrane proteins such as STIM1, Orai1 and the TRPCs have been implicated, as have their potential interactions with RyR1 [86]. The DHPR is thought to be a major contributor to ECCE [87]. STIM1 and Orai1 have been shown to colocalize to the skeletal muscle triad junction [88]. In another study, STIM1 was shown to interact with the DHPR in a Ca^2+^-independent manner and overexpression of STIM1 attenuated Ca^2+^ -release in a DHPR receptor-dependent manner suggesting that STIM1 negatively regulates Ca^2+^-release from the SR [89] and thus may be involved in both SOCE and excitation-contraction coupling. Muscle cells from the *RYR1* R163C mutant mouse exhibited elevated myoplasmic free Ca^2+^ due to a passive leak from the SR. Inhibition of non-specific plasma membrane cation channels in these cells was more effective at reducing Ca^2+^ entry and myoplasmic free Ca^2+^ than overexpression of a dominant negative Orai1. These results suggested that SOCE was not due to a STIM1/Orai1 pathway but to a non-specific plasma membrane channel, which in turn has been implicated in the MH phenotype [90]. Thus functional dysregulation associated with any one of these proteins could also affect the function of RyR1 and have implications for susceptibility to MH.

Transfecting cultured muscle cells or myotubes with one of the known causal mutations results in enhanced intracellular Ca^2+^ release when the cells are exposed to agents such as halothane, caffeine or 4-chloro-m-cresol [91–96]. Several mouse models of MH have been developed by introducing the rabbit *RYR1* cDNA into the dyspedic mouse [97], providing insights into the functional significance of introduced *RYR1* variants [98–103]. It is clear from these studies that different *RYR1* variants have different functional effects and that not every *RYR1* variant when expressed in a mouse model will exhibit a classic MH-sensitive phenotype. For example *RYR1* R163C [104] or Y522S [98] heterozygous knock-in mice exhibit symptoms like MH and are associated with increased flux of Ca^2+^ into the cytosol, while the I4898T (I4895T in mice) CCD variant causes muscle weakness, likely due to a reduction in Ca^2+^ release [105]. In addition, the Y522S homozygous mice are non-viable, while R163C and T4826I homozygous mice are viable.

### Diagnostic methods

The diagnosis of MH is based on clinical presentation or laboratory testing. The principal diagnostic features of MH are unexplained elevation of ETCO_2_ concentration, muscle rigidity, tachycardia, acidosis, hyperthermia, and hyperkalemia. The variability in the order and time of onset of signs often makes the clinical diagnosis rather difficult.

Occasionally the first indication of MH susceptibility may be a raised CK measurement. Raised CK as evidence of MH susceptibility has been previously discussed in detail [106]. Briefly, there is no clear evidence that raised CK is unequivocally symptomatic of MH susceptibility.

### Clinical grading scale

A clinical grading scale was developed by Larach and colleagues [107] through an iterative Delphic process in order to assist in clinical diagnosis. The elements of the scale are given in Table 1. Differential weighting is given to each of the manifestations of the syndrome. The scale lacks sensitivity however, since not all tests may be performed in an individual episode.

**Table 1:** Criteria used in the Clinical Grading Scale for Malignant Hyperthermia

Process	Indicator
I: Rigidity	a. Generalized muscular rigidity (in absence of shivering due to hypothermia, or during or immediately following emergence from inhalational anesthesia)
	b. Masseter spasm shortly following succinylcholine administration
II: Muscle Breakdown	a. Elevated creatine kinase >20,000 IU after anesthetic that included succinylcholine
	b. Elevated creatine kinase >10,000 IU after anesthetic without succinylcholine
	c. Cola colored urine in perioperative period
	d. Myoglobin in urine >60 μg/L
	e. Myoglobin in serum >170 μg/L
	f. Blood/plasma/serum K^+^ > 6 mEq/L (in absence of renal failure)
III: Respiratory Acidosis	a. PET_CO2_ > 55 mmHg with appropriately controlled ventilation
	b. Arterial Pa_CO2_ > 60 mmHg with appropriately controlled ventilation
	c. PET_CO2_ > 60 mmHg with spontaneous ventilation
	d. Arterial Pa_CO2_ > 65 mmHg with spontaneous ventilation
	e. Inappropriate hypercarbia (in anesthesiologist’s judgment)
	f. Inappropriate tachypnea
IV: Temperature Increase	a. Inappropriately rapid increase in temperature (in anesthesiologist’s judgement)
	b. Inappropriately increased temperature > 38.8 °C (101.8 °F) in the perioperative period (in anesthesiologist’s judgment)
V: Cardiac Involvement	a. Inappropriate sinus tachycardia
	b. Ventricular tachycardia or ventricular fibrillation

Each process is weighted and scored according to its significance in differentiating MH from other causes of change in the physiologic process. Only one element in each process need be present to qualify for scoring. A score is then generated assessing the likelihood of the episode being an MH episode on a scale from almost never to almost certain. Being a clinical scale and depending on the presence of laboratory tests, its value resides mainly in identifying those subjects with the most convincing episodes of MH for subsequent evaluation of the sensitivity and specificity of the diagnostic tests.

### Laboratory diagnostic methods

The “gold standard” for diagnosis of MH is currently an *in vitro* contracture test, which is based on contracture of muscle fibers in the presence of halothane or caffeine. Two widely used forms of this test have been developed; one (IVCT) by the European Malignant Hyperthermia group (EMHG) and the other (CHCT) by the North American Malignant Hyperthermia Group (NAMHG) [108, 109]. Using the EMHG protocol, an individual is considered susceptible to MH (MHS) when both caffeine and halothane test results are positive. An individual is considered not susceptible to MH (MHN) when both tests are negative. An individual is also diagnosed as MHS when either a positive halothane or caffeine test alone is obtained and these individuals are designated MHS(h) or MHS(c). This nomenclature was determined at the 32^nd^ EMHG meeting in Basel, Switzerland, 2013. This test is similar to the NAMHG protocol but there are differences in the concentrations used and mode of testing agents. Sensitivity of 99 % and a specificity of 94 % are obtained with the EMHG protocol [110] while figures of 97 % sensitivity and 78 % specificity are reported for the NAMHG protocol [111], which provide some confidence to the results obtained. The specificity of either protocol may be affected by neuromuscular disorders unrelated to MH, which have an associated increase in myoplasmic Ca^2+^ concentration [109, 112]. Studies based on results from monozygotic twins however, indicate that the IVCT has acceptable reproducibility [113]. A third variation of the IVCT, the caffeine skinned fiber test, does not appear to be used diagnostically outside of Japan, and has lower specificity and sensitivity than either the EMHG or NAMHG protocols [114].

IVCT is expensive, confined to specialized testing centers, requires a surgical procedure and can yield false positive or negative results. Modifications of the EMHG protocol include the use of ryanodine [115] or 4-chloro-m-cresol [116] (but to date these agents have not been included in the standard protocol). A possible alternative testing agent is the fluorinated ether sevoflurane, however trials with this agent have not found responses consistent with halothane [117].

Other biochemical, hematological and physical tests lack significant sensitivity and specificity to be used diagnostically. A further caveat with these tests is that the results may be difficult to interpret in a patient suffering from a myopathy other than MH such as Duchenne Muscular Dystrophy where intracellular Ca^2+^ is elevated at baseline.

A variety of minimally invasive diagnostic tests have been investigated. These include nuclear magnetic resonance spectroscopy to evaluate ATP depletion [118], metabolite assays and microdialysis of caffeine to elicit an enhanced release of carbon dioxide from the muscle tissue [119]. The ethics of injecting a triggering agent, even a small volume into a potentially susceptible individual have to be questioned and determination of cutoff points would be difficult.

DNA analysis, however, offers an alternative to the IVCT, requiring only a blood specimen, which can be sent to an accredited diagnostic laboratory. To date 50 to 70 % of MH susceptibility has been linked to *RYR1* with over 400 variants associated with MH being identified within this gene [120]. While the majority of variants lead to a single amino acid change in the receptor, deletions or truncations have also been reported. A number of recessive variants result in MH, CCD or related disorders [121–124].

At least 44 variants have been reported in the *RYR1* gene in association with CCD. In general terms, a single point *RYR1* variant can cause (a) CCD only, (b) MH only, (c) MH with variable CCD penetrance. In this latter case, the likelihood of an *RYR1* mutation resulting in both MH and CCD depends on a number of factors including sensitivity of mutant protein to agonists, size of the intracellular Ca^2+^ pool and the level of abnormality in channel-gating [125]. All individuals with the variant should be considered as MH susceptible, while they may or may not have CCD. If a variant specific to CCD is identified in a family, MH is not automatically excluded as a second variant may be present and MH susceptibility needs to be assessed by IVCT or CHCT or family members treated as if they are MH susceptible [126]. An MH negative parent eliminates susceptibility in the children although CCD may still be present.

While traditional DNA sequencing from either genomic DNA or complementary DNA prepared from muscle biopsy tissue are time consuming and laborious, the advent of massively parallel sequencing (or next generation sequencing, NGS) provides potentially cost effective, rapid and high throughput platforms for both variant discovery and diagnosis at the whole genome level [127]). A number of *RYR1* or *CACNA1S* variants have been identified using next generation sequencing (NGS) [128–131]. Some caution in this approach should however, be exercised as none of the currently available platforms for sequencing, or chemistry for sample preparation, or analysis software are able to yield 100 % coverage of all exons in the human genome [132]. Pathogenicity prediction is problematic (see below) and an additional consideration is the ethical dilemma associated with the reporting of incidental findings [133].

The EMHG has established criteria including functional studies of DNA variants to establish that the variant is clinically significant [134]. Thirty-four mutations within *RYR1* have been shown to cause an alteration in Ca^2+^ release from intracellular stores. A number of functional tests have been used successfully to assess the role of *RYR1* variants in Ca^2+^ release. These include the use of lymphoblastoid cell lines generated from MHS individuals [40, 135–138], COS-7 or HEK293 cells transfected with the cDNA for rabbit or human [93, 95] *RYR1* carrying point mutations introduced by site-directed mutagenesis, myotubes generated from muscle biopsy tissue and 1B5 dyspedic myotubes transduced with wild type or mutated *RYR1* cDNA [97, 139, 140]. Ca^2+^ release can be monitored and quantified directly using Ca^2+^-specific indicators or indirectly using [^3^H] ryanodine binding assays [94] or by proton release [138, 141]. Systems using 1B5 dyspedic myotubes are more physiological as they constitutively express all the components of the skeletal muscle with the exception of *RYR1* [97]. To date, all mutations functionally characterized have been shown to cause alterations in Ca^2+^ flux through the ryanodine receptor Ca^2+^ release channel.

### Pathogenicity prediction of new variants

Whole exome or targeted exon NGS is becoming the preferred option for variant detection and is being used diagnostically. The vast numbers of identified variants of unknown significance (VUS), which may or may not be associated with a certain disease have to be filtered. This is a significant bottleneck in DNA-based diagnosis for MH because of the large size of the *RYR1* gene, the large number of known uncharacterized variants and the technical difficulty involved with functional analysis. To be able to predict accurately the pathogenicity for a specific variant would considerably aid diagnosis and prevention of MH episodes.

There are many bioinformatic tools freely available (for example PolyPhen2 [142], Pmut [143], SIFT [144], MutPred [145] and SNPs&GO [146] that allow pathogenicity prediction of VUS. The accuracy of the predictions however, varies from program to program. Some of them have been trained on mutations in the on-line mendelian inheritance in man (OMIM) and human genome mutation database (HGMD) repositories, whereas others predict pathogenicity according to sequence homology of ortholog proteins.

PolyPhen2 scores are displayed in the Exome Variant Server (EVS) while both PolyPhen and SIFT scores are provided in the 1000 genomes browser. According to all the available information about a variant from the literature, genome databases as well as bioinformatic analysis and segregation analysis, the variants are classed into “definitively benign, probably benign, uncertain pathogenicity, probably pathogenic and definitely pathogenic” [7]. There is always a degree of uncertainty with any *in silico* analysis. While such predictions are useful in selecting variants for functional analysis it would be premature to begin using them for clinical diagnosis of MH susceptibility.

In summary, because of the heterogeneity of the disorder, as well as discordance within families, a negative DNA result cannot be used to rule out MH susceptibility. In addition, only those variants that have been biochemically characterized to affect SR Ca^2+^ release can be used to test for MH susceptibility.

### Differential diagnosis

A variety of unusual conditions may resemble MH during anesthesia including sepsis, thyroid storm, pheochromocytoma, and iatrogenic overheating. Hence, a high index of suspicion for these disorders as well as the ability to measure ETCO_2_ and obtain arterial and venous blood gas analysis is essential in order to differentiate them from MH. Particularly problematic is the unexplained hyperthermia following anesthesia. Since anesthetic gases generally inhibit the febrile response, the first sign of sepsis may be marked hyperthermia on emergence from anesthesia. Response to antipyretics as well as the clinical setting is often helpful in differentiating this response from MH. As stated earlier hyperthermia occurring after one hour post anesthesia is not related to MH. The differential diagnosis of unexplained increased ETCO_2_ includes hyperthermia secondary to sepsis, or iatrogenic warming, machine valve malfunction, rebreathing, as well as faulty equipment.

Outside the operating room, an MH-like syndrome may occur following injection of ionic contrast agents into the cerebrospinal fluid, cocaine overdose, and in neuroleptic malignant syndrome (NMS), serotonin syndrome and 3,4-methylenedioxy-methamphetamine (MDMA) overdose. NMS is a potentially fatal hyperthermic syndrome that occurs as a result of ingestion of drugs used in the treatment of mental and nervous conditions such as schizophrenia. The incidence is approximately 0.01–0.02 % of those being treated with these drugs such as older as well as newer antipsychotics and haloperidol, a sedative agent often used in the ICU to treat agitation. Other dopamine antagonists also have been reported to cause NMS.

The signs of NMS include muscle rigidity, acidosis, high fever and rhabdomyolysis. The pathophysiology is thought to result from dopamine receptor blockade. Treatment includes benzodiazepines, bromocriptine and even dantrolene. There does not appear to be any cross over susceptibility to MH or *vice versa*. There is no laboratory diagnostic test for the syndrome either [147, 148]. The serotonin syndrome can be associated with hyperthermia, changes in muscle tone and rhabdomyolysis in conjunction with the use of drugs that inhibit serotonin uptake or increase receptor sensitivity to serotonin. Heat-related illnesses are discussed in a later section.

If a high ionic, water-soluble radiologic contrast agent is injected intrathecally, usually as a result of drug mixup, a characteristic progression of signs occurs. After the injection, the patient appears to recover normally, but within thirty minutes involuntary jerking movements begin in the lower extremities and ascend to the upper body, finally resulting in seizures and hyperthermia. This is the result of the contrast agent entering the cerebral ventricles and requires a rapid symptomatic treatment of muscle activity, hyperthermia, and acidosis (cooling, nondepolarizing neuromuscular blockers, ventilation, and sedation [149]). The response of signs of hyperthermia, tachycardia and tachypnea to dantrolene in such syndromes is non-specific. In other words, the response to dantrolene does not *per se* prove MH susceptibility.

A syndrome often confused with MH is sudden hyperkalemic cardiac arrest during or shortly after anesthesia in young males. Following sporadic reports of such arrests, Larach and colleagues identified that patients with an occult myopathy, especially a dystrophinopathy such as Duchenne’s muscular dystrophy [150], are at risk to dramatic life-threatening hyperkalemia upon administration of succinylcholine. More recently, it has been shown that administration of potent volatile agents to such patients may produce a similar syndrome [151].

Since the most common muscular dystrophy (Duchenne’s) is found with a frequency of 1 in 3500 live male births, and the onset of symptoms of muscle weakness may be as late as 6–8 years of age, some apparently healthy children may really be at risk of succinylcholine induced hyperkalemia. Hence, when a young child or young adult experiences a sudden and apparently unexpected cardiac arrest, think of hyperkalemia, document and treat it in the standard fashion (Ca^2+^, bicarbonate, glucose and insulin, and hyperventilation). Muscle tissue should be obtained and preserved for testing for a myopathy, specifically a dystrophinopathy. In general, the patient with a dystrophinopathy that develops these anesthetic-related complications does not also exhibit classic signs of MH, such as hyperthermia or marked muscle rigidity. They do, however, develop rhabdomyolysis. Therefore, this reaction is not malignant hyperthermia *per se*, since the dystrophinopathies are caused by mutations on the X chromosome and dantrolene will not be effective.

In response to the presentation of over 30 such cases to the Food and Drug Administration Agency (FDA) of the USA in 1992, a warning was issued to avoid the use of succinylcholine in children and young adolescents for elective cases. Succinylcholine should be reserved for those cases of full stomach and possibly airway related emergencies.

Disorders not associated with MH include muscular dystrophies, myotonias, neuroleptic malignant syndrome, osteogenesis imperfecta and arthrogryposis.

### Genetic counseling

MH is an autosomal dominant genetic condition. Genetic testing has potential ramifications for the current health of that individual, but it may also have ramifications for the future health of that individual and the future health of their immediate relatives. Test results may leave the individuals disadvantaged in terms of their ability to access health insurance or life insurance, employment opportunities and, in some cultures, may even affect marital opportunities [152]. For this reason it is recommended that each individual accessing any form of genetic testing, and indeed each individual undergoing IVCT or DNA analysis, should be fully informed of all the implications of each potential result and should be able to provide informed consent prior to diagnostic testing [153].

It is also important to note that availability of the various forms of genome sequencing will place an additional burden on both the genetic counselor and the families concerned as well as the clinician ordering the test since genetic variants will sometimes be identified as an incidental finding on whole exome or whole genome testing [154]. Implications for the new born should also be considered [155].

### Interpreting risk for other family members

When initiating genetic analysis in a branch of a known family, it is important to test the individual at the highest risk first. In general, an affected proband will have inherited MH sensitivity from one of the parents. Clarification of which parent may also be MHS is useful for identifying which side of the extended family may be at risk. The risk to the siblings depends on the genetic status of the parents. If a parent is identified as MHS, then each of the proband’s siblings has a 50 % chance of also being MHS. If both parents receive an MHN result on IVCT and *RYR1* analysis – suggesting the mutation is *de novo* in the proband – then the proband’s siblings are at no greater risk than the general population. The risk for offspring of each individual with proven MHS also has a 50 % chance of being MHS. The proband’s grandchildren would be considered to be at 25 % risk until their parent’s genetic status is clarified. An individual who is MHN cannot pass MH sensitivity on to the next generation, however, if they have an affected parent, their siblings may still be at risk.

### Autonomy in clinical testing for MH

Some individuals may wish to delay IVCT or *RYR1* analysis, while they consider the information they have been given and/or make the necessary preparations. Others may decide that they do not want their risk clarified by clinical testing. These decisions should be respected and these individuals considered being MHS until proven otherwise. Care should then be taken when arranging testing for the offspring of these individuals as a positive result in the next generation will generate a result for the individual who did not want to know (the individual must have carried the gene mutation in order to pass it on).

### Management and treatment

Dantrolene is the only drug known to specifically treat MH. Dantrolene inhibits the DHPR in an RyR1-dependent manner [156], has been found to bind to a specific site on the RyR1 protein [157] and reduces RyR1 channel activity in intact muscle cells (Dirksen R – personal communication). The drug, introduced in 1979, has been responsible for lowering the mortality from MH to 1.4 % in North America (see final comment). The original preparation called Dantrium contains 20 mg of a lyophilized form of the drug per vial, which must be reconstituted before injection.

### Acute MH crisis

The essential points in the treatment of an acute MH crisis are the immediate discontinuation of trigger agents, hyperventilation, administration of dantrolene in doses of 2.5 mg/kg repeated *pro re nata* to limit MH, cooling by all routes available (intravenous saline at 4^o^ C, topical ice to all exposed areas, peritoneal exchange). Nasogastric lavage and bladder irrigation are contraindicated as complications such as gastric rupture can occur. Hyperkalaemia should be managed in a standard fashion. Ca^2+^ blockers *viz* verapamil should not be used along with dantrolene, since hyperkalemia and profound hypotension may occur with such a drug combination [16, 158]. The steps in the treatment of acute MH are shown in Table 2.

**Table 2:** Managing an MH crisis

Action	Notes
Stop potent inhalation agents	Turn vaporisers "OFF" and /or activated charcoal filters inserted into the circuit
Do not repeat succinylcholine if it has been previously administered	
Increase minute ventilation to lower ETCO_2_	Eliminate the inhalational agent
Get help	• Duty anesthestist
	• Consultant anesthetist
Prepare and administer dantrolene	• 2.5 mg/kg initial dose
	• Every 10–15 min until acidosis, pyrexia, muscle rigidity are resolving
Begin cooling measures if hyperthermic	• Tissue destruction will occur at 41.5 °C
	• Use intravenous normal saline at 4 °C.
	• Ice Packs to all exposed areas
	• More aggressive measures as needed
Stop cooling measures at 38.5 °C	
Treat arrhythmias as needed	• Amiodarone is the first choice
	• Lignocaine
	• Do not use calcium channel blockers
Secure blood gases, electrolytes, creatine kinase, blood and urine for myoglobin	• Coagulation profile check values regularly
	• Treat hyperkalemia with hyperventilation, glucose and insulin as needed
	• Once crisis is under control, an MH hotline should be contacted for further guidance
Continue dantrolene	• 1 mg/kg every 4–8 h for 24–48 h
	• Alternatively and only if recrudescence occurs, dantrolene at 2.5 mg/kg bolus
Ensure urine output of 2 mL/kg/h with	• Mannitol
	• Furosemide
	• Fluids as needed
Evaluate need for invasive monitoring and continued mechanical ventilation.	
Observe patient in Intensive Care Unit	At least 24 h
Refer patient and family for MH Testing	Contracture or DNA testing

More information on treating an MH crisis can be found on the MHANZ and MHAUS websites where detailed task cards, a management poster and other cognitive aids and educational material have been made freely available [159, 160]. Standard operating procedures for patient safety in anesthesia have also been published in the German language [161].

### Dantrolene

There are two preparations of Dantrolene available. The conventional version, Dantrium®, is available in 20 mg vials which are poorly soluble and each require 60 mL of sterile water to prepare. An average adult may therefore require 8–10 ampoules for initial treatment. Ryanodex® is a new alternative preparation approved by the FDA, available in 250 mg ampoules which only require 5 mL of sterile water diluent to reconstitute, and solubility has been improved. Therefore initial treatment can now be achieved with administration of only one ampoule. Titrate dantrolene to tachycardia and hypercarbia; there is no upper limit to the dose of dantrolene [16]. If however, more than 10 mg/kg of dantrolene is administered, the diagnosis of MH should be reconsidered. Other possible causes of MH-like symptoms include sepsis, NMS, intracranial hemorrhage, pneumonia, baclofen withdrawal [162].

Patients experiencing MH should receive dantrolene and be monitored closely for 48–72 h, since (even despite dantrolene treatment) 25 % of patients will experience a recrudescence of the syndrome [163]. Tests for disseminated intravascular coagulation (DIC) should be included as well as observation of urine for myoglobinuric renal failure. DIC is most frequent when body temperature exceeds about 41 ° C.

Since masseter muscle rigidity (MMR) may presage MH, it is most advisable to discontinue the trigger anesthetic after MMR. In an emergency, the anesthesia may continue with “non-trigger” drugs. Following MMR, patients should be admitted to an intensive care unit and monitored for signs of MH. Rhabdomyolysis occurs in virtually all patients experiencing MMR and the creatine kinase (CK) values should be checked regularly. Dantrolene should be administered if the other signs of MH occur along with MMR. Muscle biopsy for definitive diagnosis should be carefully considered.

It is remarkable that dantrolene may be efficacious in treating hyperthermia from many causes unrelated to MH with anesthesia. Based on the similarity between a variety of drug induced hyperthermic syndromes and MH, dantrolene has been used effectively to treat several other syndromes such as the neuroleptic malignant syndrome, MDMA toxicity and hyperthermia related to new onset of juvenile diabetes in adolescents [164, 165].

In many countries, a “hotline” has been established to provide emergency assistance in the management of MH. Many are listed on the web site of the Malignant Hyperthermia Association of the USA [160].

Experience from the Malignant Hyperthermia Hotline in the US as well as a recent retrospective review has shown that dantrolene may dramatically reverse life-threatening hyperthermia in a nonspecific manner. Considering that the toxicity of dantrolene is minimal when used for short periods clinicians have found the drug to be extremely useful. Adverse effects of dantrolene in short term administration are minor and may include phlebitis in 9 % of cases, transient muscle weakness in 21 %, gastrointestinal upset in 4 % and respiratory compromise in patients with preexisting muscle disorders [166]. A caveat is that success in controlling hyperthermia does not imply that the patient is at risk for Malignant Hyperthermia Syndrome.

### Management of the MH susceptible patient for anesthesia

Ideally the patient should be seen preoperatively and risks discussed. In most cases the risk of problems is low and the possibility of a stress-induced episode can effectively be regarded as zero.

Patients who are known to be MH susceptible may be anesthetized with regional anesthesia or local anesthesia without problems. If general anesthesia or sedation is required, potent volatile agents and succinylcholine must be avoided. Non-depolarizing muscle relaxants and all intravenous inducing agents are safe to use. Laryngeal mask airways are safe to reuse if an idle period of 15 h [167] has been observed but the major use of these airways is now single use.

Preparation of newer generation anesthetic machines has become complex. Silicone products incorporated into these machines absorb inhalational anesthetics and result in prolonged release of the agent. Flushing of these machines can take longer that 60 min to achieve a safe level of agent [168]. A vapor-free anesthetic machine would eliminate this problem but it is likely that most anesthetic departments do not have such a machine available. Recent research has demonstrated that activated charcoal filters reduce anesthetic concentrations to safe levels within several minutes and are now being used in some countries. Advice on flow rates should be adhered to [169, 170]. Vaporizers should be disabled, drained or removed if possible.

While traditionally, MH susceptible patients who have undergone non-triggering anesthesia were monitored routinely for four hours in the post-anesthesia care unit, this practice is no longer thought to be necessary [171]. Pretreatment with dantrolene is also not necessary.

### Preventive measures

Preventative measures include preoperative assessment and identification of an inherited association with a known family, managing a patient with a suspected history as MH susceptible until testing is undertaken, an operating theatre list of susceptible names in the community and an indication of MH susceptibility on the anesthetic record audit form, labeling hospital records together with a national alert warning on records, and family education is helpful.

Patients with any form of muscle disorder should not receive succinylcholine and caution should be exercised with administration of inhalational agent to patients with other muscle disorders particularly muscular dystrophies especially hypokalemic periodic paralysis, CCD, Duchenne or Becker.

All patients receiving more than a brief general anesthetic should have their core temperature monitored.

Young patients (below age 12 approximately) should not receive succinylcholine for elective procedures, in order to avoid the possibility of hyperkalemic response in a patient with undiagnosed muscular dystrophy.

### Total intravenous anesthesia

While it is important to avoid inhalational anesthetics for individuals susceptible to MH, total intravenous anesthesia (TIVA) is not universally recommended for individuals who are not susceptible to MH. Choice of anesthesia, however, is in the realm of the clinician involved. Anesthetic vaporizers and anesthetic machines including gas analyzers are universally available whereas the equipment required for TIVA are not, particularly in developing countries. Inhalational anesthesia is quick, painless and does not require intravenous access, considerations of importance in emergency situations and in children. TIVA carries a higher risk of awareness (5-10x as high) than volatile anesthesia because the amount of anesthetic agent in the patient’s body cannot be measured. Routine use of awareness monitoring is recommended for TIVA general anesthetics [172]. Prolonged inhalational anesthesia has been shown to be safe, the depth of anesthesia can be readily quantified and steady-state measures of potency have been determined for all inhalational anesthetics [173].

## Unresolved issues

### Risk factors

#### Stress and exercise

In 1966 the Porcine Stress Syndrome was identified as an “awake” MH episode. Stresses such as fighting cause a rapid death in these animals. Exercise and heat-stroke as potential triggers for an MH episode continue to be debated. Gronert and Denborough, both reported patients with “awake” MH episodes, the latter being patients with exercise-induced heat stroke who responded to dantrolene [174–176]. Perhaps the most convincing, though unfortunate, episode of exercise-induced MH was reported by Tobin et al., a fatal episode in a 13-year-old boy who had experienced a clinical episode of MH and developed signs of MH following exercise some months later. He and other family members were found to have a causative *RYR1* mutation [177]. Brown et al. reported a possible viral trigger [178]. Several more recent reports also link MH to exertional heat-stroke [179–181]. Fatal drug-free stress-induced MH in two unrelated children was also recently linked to the presence of variants in *RYR1*. Expression of *RYR1* with these variants in an heterologous system indicated hypersensitivity to RyR1 agonists, consistent with “awake” MH and heat sensitivity [182].

Further physiological evidence of stress-related MH has been demonstrated by pH changes in MHS muscle recovering from violent exercise [183]. The sympathetic nervous system appears to be only secondarily involved [184]; serotonin (5-hydroxytyrosine) agonists may cause an MH-like syndrome in susceptible pigs but there is limited support for serotonin as a trigger in stress induced episodes [185, 186]. Recent research in mice with the human *RYR1* Y522S^1^ mutation indicates abnormal sensitivity to increased environmental temperatures associated with abnormal Ca^2+^ release [98]. This latest report, however, should be considered with some caution as the homozygous Y522S mutation in mouse is embryonic lethal, which is a different phenotype to that observed with the homozygous *RYR1* R615C^2^ mutation in pigs and the small number of homozygous *RYR1* variants in humans which clearly do not cause embryonic lethality. A more recent study however showed that mice heterozygous for the Y522S mutation exhibited attenuated thermal sensitivity after eccentric exercise [187]. Another study, however, reports that a “knock-in” mouse heterozygous for the human *RYR1* R163C mutation is more representative of the human phenotype and thus may provide an important model system for further study of awake-MH [104]. Heat stress also triggers fulminant MH in mice expressing the rabbit equivalent of the human *RYR1* T4826I mutation [188].

Wappler et al. described a 34-year-old male with recurrent fever, fatigue, muscle cramping, and aching with mild exercise and emotional stress [189]. IVCT demonstrated an MHS response and a “causative” mutation. Others have reported similar findings [190] and Wappler also reported a series of individuals with positive IVCT and DNA tests [191]. Cappachione et al. described a patient with exercise-induced rhabdomyolysis (ER) and multiple loci variants [64]. A possible conclusion is that a small subset of MH patients may display muscle damage and perhaps more ominous signs with exercise or other stresses. It is recommended that MH is excluded in patients who have had episodes of exertional heat stroke [192]. Despite possible links between exertional heat stroke and MH however, treatment with dantrolene has not been rigorously examined.

The risk of an exercise-induced event is remote and patients should be advised to continue with a normal lifestyle although patients should be cautioned regarding the remote, but conceivable possibility of heat stroke in environments in which exposure to high heat and humidity is possible.

### Statin therapy

It has been suggested that statins can affect MHS muscle responses as positive contracture results, using the European MH Group protocol, have been observed in some patients on statin therapy [193]. Vladutiu et al. investigated 197 individuals with severe statin myopathy and compared the group with 2 other groups (1) 163 subjects with mild statin myopathy and (2) 122 patients in a statin-tolerant group. *RYR1* variants were identified in 3 severe statin myopathy cases, 1 mild myopathy statin individual, 8 patients with non-drug-induced myopathy and no variants were present in controls. This study may indicate that statins may unmask underlying serious myopathies [194].

### Discordance

Given the confidence provided by functional analysis of *RYR1* variants, the problem of discordance between *RYR1* mutations and MHS and MHS (h) or MHS (c) still remains the largest problem associated with genetic diagnosis of susceptibility to MH. The MHS (h) or MHS (c) diagnosis is the most problematic and exhibits a much higher level of discordance than does MHS. Correlation between *RYR1* variants and IVCT is greater for the caffeine (c) than the halothane (h) response [195] suggesting that the MHS(c) has greater diagnostic potential. The NAMHG protocol does not allow the MHS (h) or MHS (c) diagnosis; the potential for discordance between IVCT phenotype and *RYR1* genotype is therefore much greater. In a large UK study investigating the relationship between *RYR1* genotype and IVCT phenotype, discordance was identified in seven families (nine individuals), with five false-positives and four false-negatives [196]. Discordance has also been observed between *RYR1* causative mutations and IVCT results in 6/96 individuals in 4 Belgian families [197]. *RYR1* mutation negative MHS individuals have also been observed (Recent unpublished data give an approximate 2.5 % discordance rate of this type in a large series of UK patients.) Clear evidence of the involvement of genes as well as *RYR1*, has been shown in a New Zealand Maori pedigree where MHS correlates with a *RYR1* T4826I mutation [178] but three branches of the family possess unrelated chromosome 19 haplotypes, without the T4826I mutation in unambiguous MHS individuals spanning three or four generations. While some discordance may be explained by the existence of other yet unidentified variants, false positive IVCT tests [198] and variants associated with weak contractures have also been implicated. Discordance has been attributed to epigenetic alterations at the *RYR1* locus causing silencing [199] but until recently no evidence had been provided that the *RYR1* gene would be silenced [200]. A more recent report however, suggests that decreased expression of muscle-specific microRNAs correlated with epigenetic changes at the *RYR1* locus and reduced expression of *RYR1* because of gene silencing [201]. Taken together, these observations suggest that DNA testing should always be used in selected, genetically characterized families, as well as within the guidelines for DNA testing identified by the EMHG or MHAUS [202–204]. Using both IVCT and genetic diagnosis, a higher proportion of true positives are likely to be identified than by simply relying on one or other test.

### Resources

Many anesthesia textbooks, web sites and articles contain very thorough descriptions of MH and related syndromes. However, these sources often fail to provide information for patients (patient-specific information). Various voluntary organizations throughout the world are dedicated to assisting patients, physicians, anesthesia providers of all types and any one else in managing the MH susceptible and keeping these individuals up to date with the latest information regarding MH.

In the United States, the Malignant Hyperthermia Association of the United States (MHAUS) provides newsletters, printed information, an informative website [204] to meet the needs of the various groups interested in MH. In addition, a hotline provides direct consultation for providers in real time management of MH episodes or questions related to specific patients as to their likelihood of developing MH and the optimum management of an episode. MHAUS, similar to other MH patient advocacy organizations is not for profit supported by voluntary contributions. The North American MH Registry supports a patient-specific database with detailed information as to the phenotypic presentations as well as diagnostic test results. The Registry is a subsidiary of MHAUS and is located at Children’s Hospital of Pittsburgh [205].

The European MH group [134] coordinates testing procedures throughout Europe and is made up of professionals investigating MH. Patient supported MH associations exist in France, Germany, Switzerland, Japan, United Kingdom and several other countries. In South Africa, issues related to MH are subsumed under the Muscular Dystrophy Association of that country. These organizations have been crucial to the education of anesthesia providers in diagnosing and managing MH and helping patients better understand the disorder.

## Conclusions

MH remains a serious risk factor for susceptible individuals undergoing general anesthesia using volatile agents. A number of environmental stresses have also been implicated as risk factors in MHS individuals but there is as yet no clear consensus from the literature. While two genes have been unequivocally linked to causation of MH, discordance exists and the potential for the involvement of other genes cannot be discounted. The incidence of death due to MH has decreased in the last thirty years but at the same time the prevalence of genetic variants in the general population has been estimated to be much higher than was originally thought. In addition, unresolved issues including discordance, “awake” MH and the influence of statin therapy suggests that genetic variants previously associated mainly with anesthetic-induced MH may have a much wider range of pathological phenotypes. As a final comment, mortality in MH has been reduced from 80 % to 1.4 % [206] although a recent report shows a further increase [23] so there is still a significant mortality from this disorder and vigilance must be maintained with any anesthetic where triggering drugs are administered.

## Abbreviations

1B5: Cell line isolated from the *RYR1* null mouse
ACTA1: Gene encoding alpha actin
ATP: Adenosine Triphosphate
C: Cysteine
Ca^2+^: Calcium ion
*CACNA1S*: Gene encoding α1 s subunit of the dihydropyridine receptor
*CASQ1*: Gene encoding type 1 calsequestrin
CCD: Central Core Disease
cDNA: Complementary deoxyribonucleic acid
CHCT: Caffeine Halothane Contracture Test
CIC-1: Skeletal muscle chloride channel
CK: Creatine Kinase
COS7: Cell line derived from the African Green Monkey
DIC: Disseminated Intravascular Coagulation
DHPR: Dihydropyridine receptor
DNA: Deoxyribonucleic acid
ECCE: Excitation-coupled calcium entry
ER: Exercise-induced rhabdomyolysis
EMHG: European Malignant hyperthermia Group
ETCO_2_: End-tidal Carbon Dioxide
EVS: Exome Variant Server
FDA: Food and Drug Administration Agency
HEK: Human Embryonic Kidney
HGMD: Human Gene Mutation Database
I: Isoleucine
ICU: Intensive Care Unit
IVCT: *In Vitro* Contracture Test
*JSPR1*: Gene encoding Junctional Sarcoplasmic Reticulum Protein 1 (JP-45)
MDMA: 3,4-methylenedioxy-methamphetamine
MH: Malignant hyperthermia
MHAUS: Malignant Hyperthermia Association of the United States
MHANZ: Malignant Hyperthermia Australia and New Zealand
MHN: Not susceptible to Malignant hyperthermia
MHS: Malignant hyperthermia susceptible
Min: minute/s
MmD: Multiminicore myopathy
MMR: Masseter Muscle Rigidity
MutPred: Mutation Prediction
MYH7: Gene encoding myosin heavy chain 7
NAMHG: North American Malignant Hyperthermia Registry
NGS: Next Generation Sequencing
NMS: Neuroleptic Malignant Syndrome
OMIM: Online Mendelian Inheritance in Man
*Orai1*: Gene encoding ORA1 calcium release-activated calcium modulator 1
PolyPhen: Polymorphism Prediction
Pmut: Pathogen Mutation Prediction
SERCA: Sarco/endoplasmic reticulum calcium ATPase
SIFT: Sorting Intolerant From Tolerant
SOCE: Store-operated calcium entry
SNPS&GO: Single Nucleotide Polymorphisms and Gene Ontology
SR: Sarcoplasmic Reticulum
SRP-27: Stress response protein 27
R: Arginine
*RYR1*: Gene encoding ryanodine receptor 1
RyR1: Ryanodine receptor protein
S: Serine
*STAC3*: Gene encoding the SH3 and cysteine rich domain 3 protein
*STIM1*: Gene encoding stromal interacting protein 1
T: Threonine
TIVA: Total intravenous anesthesia
TRPC: Transient Receptor Potential Channels
UK: United Kingdom
USA: United States of America
VUS: Variant of Unknown Significance
Y: Tyrosine

## Footnotes

1 Human *RYR1* mutations are numbered according to NP_000531.2, GenBank.

2 Porcine RYR1 mutations are numbered according to NP_001001534.1, GenBank.
